# Selecting SNPs informative for African, American Indian and European Ancestry: application to the Family Investigation of Nephropathy and Diabetes (FIND)

**DOI:** 10.1186/s12864-016-2654-x

**Published:** 2016-05-04

**Authors:** Robert C. Williams, Robert C. Elston, Pankaj Kumar, William C. Knowler, Hanna E. Abboud, Sharon Adler, Donald W. Bowden, Jasmin Divers, Barry I. Freedman, Robert P. Igo, Eli Ipp, Sudha K. Iyengar, Paul L. Kimmel, Michael J. Klag, Orly Kohn, Carl D. Langefeld, David J. Leehey, Robert G. Nelson, Susanne B. Nicholas, Madeleine V. Pahl, Rulan S. Parekh, Jerome I. Rotter, Jeffrey R. Schelling, John R. Sedor, Vallabh O. Shah, Michael W. Smith, Kent D. Taylor, Farook Thameem, Denyse Thornley-Brown, Cheryl A. Winkler, Xiuqing Guo, Phillip Zager, Robert L. Hanson, the FIND Research Group

**Affiliations:** Phoenix Epidemiology and Clinical Research Branch, National Institute of Diabetes and Digestive and Kidney Diseases, National Institutes of Health, Phoenix, AZ 85014 USA; Genetic Analysis and Data Coordinating Center, Case Western Reserve University, Cleveland, OH 44104 USA; Division of Nephrology, The University of Texas Health Science Center, San Antonio, TX 78229 USA; Department of Nephrology, Harbor-UCLA Medical Center, Torrance, CA 90502 USA; Wake Forest School of Medicine, Winston-Salem, NC 27157 USA; National Institute of Diabetes and Digestive and Kidney Diseases, Bethesda, MD 20892 USA; Welch Center for Prevention, Epidemiology, and Clinical Research, Baltimore, MD 21205 USA; The University of Chicago Medical Center, Chicago, IL 60637 USA; Loyola University Medical Center, Chicago, IL 60153 USA; Divisions of Nephrology and Endocrinology, David Geffen School of Medicine at UCLA, Los Angeles, CA 90095 USA; Division of Nephrology and Hypertension, Department of Medicine, UC Irvine School of Medicine, University of California, Orange, 92868 CA USA; Hospital for Sick Children, University Health Network and the University of Toronto, Ontario, M5G1X8 Canada; Institute for Translational Genomics and Population Sciences, Los Angeles Biomedical Research Institute and Department of Pediatrics, Harbor-UCLA Medical Center, Torrance, CA 90502 USA; Departments of Medicine and Physiology and Biophysics, Case Western Reserve University, Cleveland, OH 44104 USA; The University of New Mexico, Albuquerque, NM 87131 USA; National Human Genome Research Institute, NIH, Bethesda, MD 20892 USA; Department of Biochemistry, Faculty of Medicine, Kuwait University, Kuwait City, Kuwait; The University of Alabama at Birmingham, Birmingham, AL 35233 USA; Center for Cancer Research, National Cancer Institute, NIH, Leidos Biomedical, Inc., Frederick National Laboratory for Cancer Research, Frederick, MD 21702 USA

**Keywords:** Individual genetic ancestry, Population structure, SNP, Diabetic nephropathy

## Abstract

**Background:**

The presence of population structure in a sample may confound the search for important genetic loci associated with disease. Our four samples in the Family Investigation of Nephropathy and Diabetes (FIND), European Americans, Mexican Americans, African Americans, and American Indians are part of a genome- wide association study in which population structure might be particularly important. We therefore decided to study in detail one component of this, individual genetic ancestry (IGA). From SNPs present on the Affymetrix 6.0 Human SNP array, we identified 3 sets of ancestry informative markers (AIMs), each maximized for the information in one the three contrasts among ancestral populations: Europeans (HAPMAP, CEU), Africans (HAPMAP, YRI and LWK), and Native Americans (full heritage Pima Indians). We estimate IGA and present an algorithm for their standard errors, compare IGA to principal components, emphasize the importance of balancing information in the ancestry informative markers (AIMs), and test the association of IGA with diabetic nephropathy in the combined sample.

**Results:**

A fixed parental allele maximum likelihood algorithm was applied to the FIND to estimate IGA in four samples: 869 American Indians; 1385 African Americans; 1451 Mexican Americans; and 826 European Americans. When the information in the AIMs is unbalanced, the estimates are incorrect with large error. Individual genetic admixture is highly correlated with principle components for capturing population structure. It takes ~700 SNPs to reduce the average standard error of individual admixture below 0.01. When the samples are combined, the resulting population structure creates associations between IGA and diabetic nephropathy.

**Conclusions:**

The identified set of AIMs, which include American Indian parental allele frequencies, may be particularly useful for estimating genetic admixture in populations from the Americas. Failure to balance information in maximum likelihood, poly-ancestry models creates biased estimates of individual admixture with large error. This also occurs when estimating IGA using the Bayesian clustering method as implemented in the program STRUCTURE. Odds ratios for the associations of IGA with disease are consistent with what is known about the incidence and prevalence of diabetic nephropathy in these populations.

**Electronic supplementary material:**

The online version of this article (doi:10.1186/s12864-016-2654-x) contains supplementary material, which is available to authorized users.

## Background

The Family Investigation of Nephropathy and Diabetes (FIND) is a multicenter study that is designed to find genes that contribute to the onset of diabetic nephropathy in four target, self-reported, heritage groups: European Americans, Mexican Americans, American Indians, and African Americans [1–4]. Two strategies were employed to ascertain the role of specific genes, a family-based linkage study and a case–control genome-wide association study (GWAS). In the GWAS each person in the four groups was typed for 1 M single nucleotide polymorphisms (SNPs) on a common platform after which the genotype distributions in the cases and controls for each SNP were compared to identify risk alleles with genome-wide significance. A common practice in GWAS such as the FIND is to control for population stratification by adding principal components (PCs) or individual genetic ancestry (IGA) estimates as covariates to the statistical models [5].

While the assessment of IGA is potentially important for GWAS and for other genetic analyses, the evaluation of an American Indian heritage has been difficult because there has been little information on ancestry informative markers (AIMs) from a large sample of American Indians typed on a commercially available platform. The Pima Indians of the Gila River Indian Community in Arizona, who have a very high prevalence of type 2 diabetes, are one of the most intensively studied American Indian groups in the United States; genetic and heritage analyses have been performed in this native group for many years, involving research that includes GWAS with 100 K and 1 M SNP arrays [6–10]. Pima Indians also constituted a large proportion of the American Indian sample in the FIND. Therefore data from the Pima Indian GWAS, conducted with the Affymetrix Genome-Wide Human 6.0 SNP array [11], were used to isolate informative markers for IGA in American Indians, which were then combined with 3 populations from HapMap to create a panel of AIMs.

**Fig. 1:**
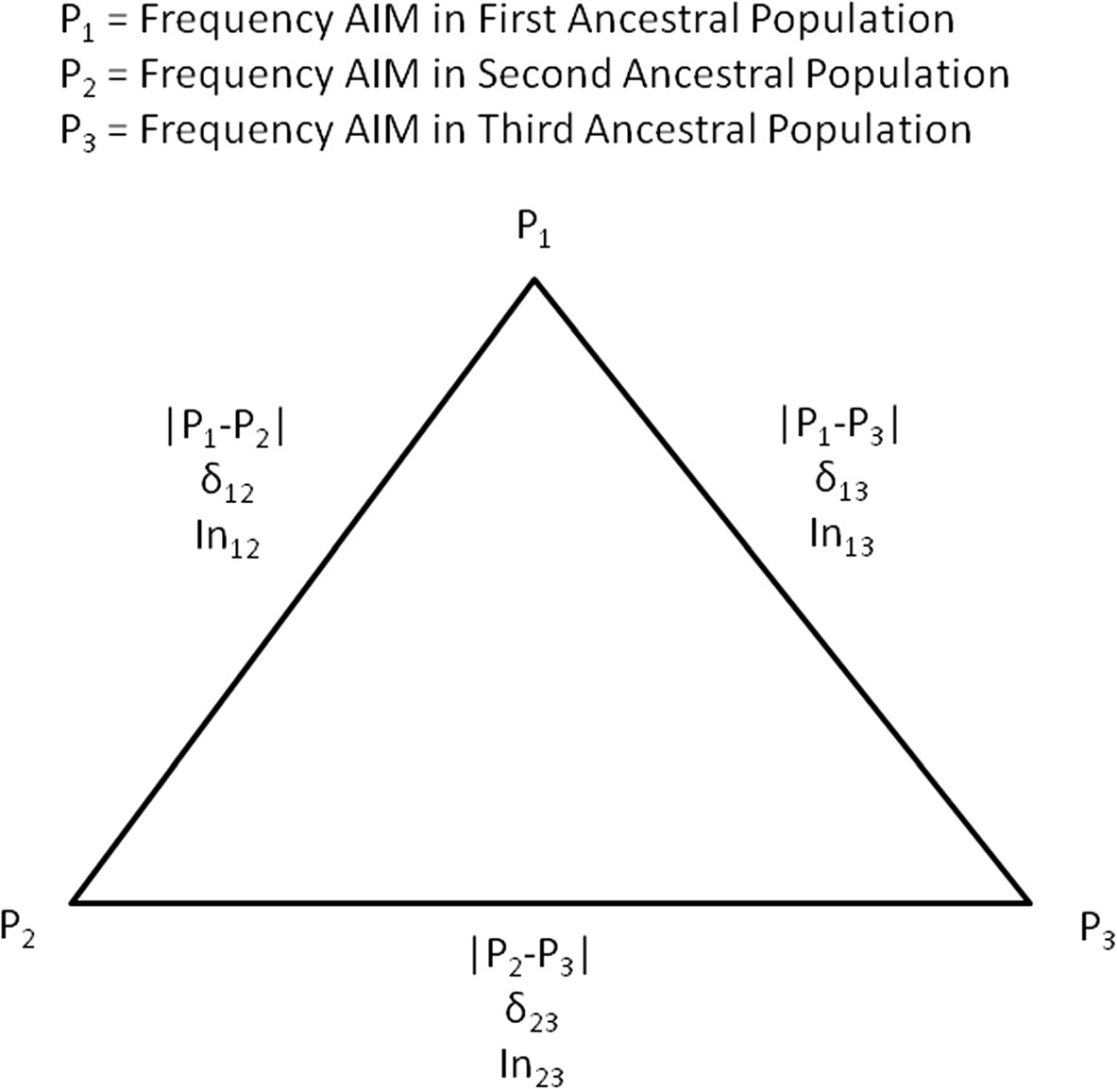
Information contrasts. For a 3-ancestral population model there are three information contrasts that are represented by the absolute value of the difference of the respective allele frequencies for allele 1 of the SNP: |P_1_-P_2_|, |P_1_-P_3_|, and |P_2_-P_3_|, a value that is usually given the symbol δ. The variable *In* is the information-for-assignment statistic. Accurate individual ancestry estimates depend upon balancing the information between these 3 contrasts

We use the AIMs and FIND samples to address an important methodological issue, that in poly-ancestry (>2) maximum likelihood models the accuracy of the estimates depends on balancing the information contrasts among the ancestral populations. [We define an information contrast (In) as the information in the difference (δ) in SNP allele frequency between a pair of ancestral populations (Fig. 1).] The problem was revealed in the course of this research when preliminary analyses involving 1390 SNPs returned apparently incorrect IGA estimates, and we suspected that this was due to insufficient information regarding one of the contrasts between ancestral populations. In order to explore the reason for this discrepancy we first defined three allele frequency contrasts in a 3 ancestry model with European (EU), American Indians (AI), and African (AF) as ancestral populations: |EU-AI|, |EU-AF|, and |AI-AF|. Then we chose subsets of 1300 AIMs that maximized the information for each contrast--450, 450, and 400 SNPs, respectively--and used these individually and together to estimate individual ancestry in the respective ancestral populations from HapMap and the Pima, in which one expects the mean ancestral component to approximate 1.0; e.g., the expectation for the Pima is that mean AI ancestry will approximate 1.0. The origin of the unstable estimates was traced to a set of SNPs in which the information is not balanced across the 3 contrasts. We define a balanced model as one that includes markers that provide suitable information for contrasting all pairs of parental populations and show that, when the model is not balanced, the IGA estimates are incorrect with large error. The method and results are presented below.

## Methods

### Study participants and phenotypes

The criteria for diabetes, nephropathy, and the overall study design for the FIND have been previously described [1–4]. The FIND is a multi-ethnic family study of severe kidney disease, where the index case had diabetic nephropathy and at least one sibling reported a diagnosis of either diabetic nephropathy or long-standing diabetes without nephropathy. Samples from four different ethnic FIND groups were collected: African American, American Indian, European American, and Mexican American. For the discovery GWAS unrelated cases and controls were genotyped, yielding one individual per pedigree, except that in American Indians and Mexican Americans, because the total available sample was small, some family members were also genotyped. Patients with severe DN based upon diabetes duration > 5 years and urine albumin/creatinine ratio (UACR) ≥ 0.3 mg/g or with severe kidney disease (ESRD) were defined as cases. Controls had DM durations ≥ 9 years, UACR < 30 mg/g, and serum creatinine < 1.6 mg/dl (males) or < 1.4 mg/dl (females) without first-degree relatives having kidney disease. Additional cases and controls that were not part of the original FIND study were included to increase the statistical power.

### Genotyping

A total of 5156 discovery DNA samples, plus 244 blind duplicates, was submitted to Affymetrix, Inc. (Santa Clara, CA) for genotyping. Genotypes were generated with the Affymetrix Genome-Wide Human 6.0 SNP array [11] using the Affymetrix Commercial Service (Santa Clara, California), via a contract to Translational Genomics Research Institute (TGEN, Phoenix, AZ). Samples were submitted at a concentration of 100 ng/μl in Tris-EDTA buffer, then plated according to ethnic membership that included HapMap controls and blind duplicates on each plate. Samples were tested for DNA quality and quantity using PicoGreen prior to genotyping. All ethnic groups were genotyped with the Affymetrix 6.0 chip during the GWAS phase. Genotypes were called using the Birdseed version 2 algorithm [12] implemented in the Genotyping Console software (Affymetrix). Alleles 1 and 2 for each SNP are assigned in the order that they are found in the HapMap data set.

### Statistical methods

Estimates of IGA and their variances were calculated for each subject in a three parent population model by a fixed parental allele maximum likelihood method [13]. A likelihood function *L (μ*_*1*_*, μ*_*2*_*, μ*_*3*_*)* is maximized with respect to the population parameters for European (EU, *μ*_*1*_), American Indian (AI, *μ*_*2*_), and African heritage (AF, *μ*_*3*_), giving respective statistics *m*_*1*_, *m*_*2*_, and *m*_*3*_, in the interval [0, 1]. The likelihood algorithm maximizes *m*_*1*_ and *m*_*2*_ based on *G* SNPs. Let p_ijg_ be the frequency of the *j*^*th*^ allele for the *g*^*th*^ SNP with codominant alleles *A1* and *A2* in the *i*^*th*^ ancestral population for which Europeans are ancestral population i = 1, American Indians i = 2, and Africans i = 3. Then let Δ_g_ be defined as the allele frequency difference for the *g*^*th*^ SNP where:

$$ \varDelta {1}_g={p}_{11g}-{p}_{31g} $$

$$ \varDelta {2}_g={p}_{21g} - {p}_{31g} $$

$$ \varDelta {3}_g={p}_{12g} - {p}_{32g} $$

$$ \varDelta {4}_g={p}_{22g} - {p}_{32g}. $$

For the *g*^*th*^ SNP and ancestral proportions *m*_*1*_*and m*_*2*_ the allele frequencies *P*_*hA1*_ and *P*_*hA2*_ in the hybrid population can be estimated by

$$ {P}_{hA1g} = {p}_{31g} + {m}_1\varDelta {1}_g + {m}_2\varDelta {2}_g $$

$$ {P}_{hA2g} = {p}_{32g} + {m}_1\varDelta {3}_g + {m}_2\varDelta {4}_g. $$

Under Hardy-Weinberg equilibrium the likelihoods for the three genotypes at SNP *g* are:

$$ L{(A1A1)}_g = \ln \left({\left({P}_{hA1g}\right)}^2\right) $$

$$ L{(A1A2)}_g = \ln \left(2{P}_{hA1g}{P}_{hA2g}\right) $$

$$ L{(A2A2)}_g = \ln \left({\left({P}_{hA2g}\right)}^2\right). $$

When calculating one likelihood *L*_*g*_ for *G* genotypes, for each possible combination of *m*_*1*_ and *m*_*2*_ ancestral proportions (in increments of 0.001), the estimates (ml*m*_1_, ml*m*_2_) are those which maximize the likelihood, that is:

$$ { \max}_{\begin{array}{c}\hfill {m}_1=0\to 1\hfill \\ {}\hfill {m}_2=0\to 1\hfill \end{array}}{\displaystyle {\sum}_{g= 1}^G{L}_g,} $$

and

$$ ml{m}_3=1.0-\left( ml{m}_1 + ml{m}_2\right). $$

The variances and covariance are calculated as (See Appendix for derivation of the information matrix) [14]:

$$ {\left[\begin{array}{cc}\hfill {\displaystyle \sum_{g=1}^G}{I}_g^{m_1}\hfill & \hfill {\displaystyle \sum_{g=1}^G}{I}_g^{m_1{m}_2}\hfill \\ {}\hfill {\displaystyle \sum_{g=1}^G}{I}_g^{m_1{m}_2}\hfill & \hfill {\displaystyle \sum_{g=1}^G}{I}_g^{m_2}\hfill \end{array}\right]}^{-1}=\left[\begin{array}{cc}\hfill V\left( ml{m}_1\right)\hfill & \hfill Cov\left( ml{m}_1, ml{m}_2\right)\hfill \\ {}\hfill Cov\left( ml{m}_1, ml{m}_2\right)\hfill & \hfill V\left( ml{m}_2\right)\hfill \end{array}\right] $$

$$ V\left( ml{m}_3\right) = V\left( ml{m}_1\right) + V\left( ml{m}_2\right)+2Cov\left( ml{m}_1, ml{m}_2\right) $$

with standard error

$$ SE\left( ml{m}_i\right)=\sqrt{V\left( ml{m}_i\right)}. $$

Populations from the HapMap were chosen to represent European and African origins [15]; genotypes for SNPs represented on the Affymetrix array were obtained from the HapMap website for inclusion as parental AIMs. To represent Europe, CEPH “Centre d’Etude du Polymorphisme Humain”, Utah residents (*N* = 174) with ancestry from northern and western Europe (HapMap abbreviation: CEU) were used; allele frequencies were represented by P_EU_ and ancestry by EU. To represent Africa, the allele frequencies for the Yoruba (*N* = 209) in Ibadan, Nigeria (HapMap abbreviation: YRI) and the Luhya (*N* = 110) in Webuye, Kenya (HapMap abbreviation: LWK) were averaged when the SNP was present in both populations, or used singly from either group when present in just one; allele frequency is represented as P_AF_ and ancestry as AF. To represent American Indians (AI), Pima Indians in Arizona were chosen with genotypic data from individuals who had participated in a GWAS conducted with the Affymetrix 6.0 array (*N* = 964) [7, 8]. Each person in this sample was a self-reported, full heritage Piman (Pima or Tohono O’odham or combination of the two tribes), allele frequency is represented as P_AI_ and ancestry as AI. Informative loci were identified across the 22 autosomes. Each of the three allele frequency differences, δ, contrasts for allele 1, |P_EU_-P_AI_|, |P_EU_-P_AF_|, |P_AI_-P_AF_|, is represented by a set of markers such that one contrast was maximized for information, δ ≥ 0.5, while δ < 0.3 for the other two (Fig. 1, Additional Files 1, 2, and 3). SNPs were selected such that within each set there is at least 500 kb distance between syntenic SNPs; thus, linkage disequilibrium among SNPs is expected to be minimal. SNPs with alleles A/T and C/G were not included because of the ambiguity in their interpretation. After the first selection of AIMS the 128 American Indians who were common to the FIND study and the Phoenix GWAS parental group were compared for each informative SNP. A replicate typing error threshold ≤ 0.032 was established for inclusion of a SNP. To help balance the information between the 3 sets of AIMs the information-for-assignment statistic [16]

$$ {I}_n = {\displaystyle \sum_{j=1}^G}\left(-{p}_j^l\  log\ {p}_j^l + {\displaystyle \sum_{i=1}^K}\frac{p_{ij}}{K} log{p}_{ij}\right) $$

was used, where *p*_*ij*_ is the frequency of allele 1 at the *jth* SNP in the *ith* ancestral population, and *p*_*j*_^*l*^ is the overall average frequency of allele 1 at SNP *j*. In addition, *F*-statistics were calculated by the method of Weir and Cockerham [17] to determine the utility of *F*_*st*_ for balancing information in the contrasts.

Estimates of individual admixture were also calculated for the 4 parental samples with the STRUCTURE [18] program to compare this Bayesian clustering method with the fixed parental allele algorithm and to determine whether either or both were vulnerable to the unbalanced information in the choice of human ancestry SNPs.

Principal components (PCs) were computed using SNPs that passed quality control and were not in genomic regions with extended linkage disequilibrium (LD). Specifically, markers in the following regions were excluded: chromosomes 5 (44–51.5 Mb), 6 (25–33.5 Mb), 8 (8–12 Mb), 11 (45–57 Mb), and 17 (40–43 Mb). The PC analysis was computed on the combined ethnic samples for the GWAS. The first two principal components were determined to account for a large proportion of the genetic variation in the multi-ethnic PC analysis and appropriately reduce the inflation factor in the ethnic-specific logistic regression models. Outlying individuals based on the first two PCs were excluded from the GWAS and, thus, are not included in the present analysis. A total of 33 individuals were omitted based on outlying PCA values.

Logistic regression was performed by standard methods with the disease, diabetic nephropathy, as the dependent variable and enrolment age, sex (women), enrolment center, and the respective heritage estimates as explanatory variables.

## Results

Across the 22 autosomes 1300 SNPs were selected as informative for individual ancestry with δ ≥ 0.5 (Table 1). The number of informative SNPs generally scaled with the size of the chromosome. Three distinct sets of SNPs were chosen such that each set was maximized for its information in one of the three contrasts, while the three sets together were balanced for information across the three contrasts. There were 450 SNPs in each of the contrasts |P_EU_-P_AI_| and |P_EU_-P_AF_|, and 400 SNPs maximized for information in the contrast |P_AI_-P_AF_|.

**Table 1:** Descriptive statistics for 1300 ancestry informative SNP Loci

			Maximized contrasts, δ ≥ 0.5		
Chromosome	#SNPs	Mean distance (Bp)	|P_EU_-P_AI_| *N* = 450	|P_EU_-P_AF_| *N* = 450	|P_AI_-P_AF_| *N* = 400
1	113	4,747,709	44	34	35
2	101	4,296,466	35	36	30
3	89	4,427,268	29	30	30
4	89	4,480,508	26	32	31
5	87	4,286,121	29	33	25
6	76	4,328,735	33	27	16
7	84	3,812,870	25	34	25
8	85	3,480,996	27	33	25
9	66	4,917,071	23	25	18
10	59	4,358,358	17	24	18
11	66	4,356,288	32	14	20
12	51	4,784,074	17	20	14
13	54	3,707,021	15	19	20
14	36	5,120,754	11	12	13
15	50	3,516,390	14	19	17
16	49	3,846,889	22	12	15
17	29	4,218,684	8	11	10
18	34	3,410,019	13	11	10
19	19	6,022,778	6	8	5
20	31	4,143,198	10	8	13
21	15	3,683,800	6	5	4
22	17	4,038,881	8	3	6

The power of each SNP to estimate IGA is proportional to the magnitude of the allele frequency difference between the two parental populations, or δ, in the three difference-contrasts for each marker, |P_EU_-P_AI_|, |P_EU_-P_AF_|, and |P_AI_-P_AF_|, and the information-for-assignment statistic *In*, which was also calculated for each contrast (Table 2). Within each information contrast, and its set of SNPs, the two statistics are closely matched with δ ≅ 0.53 for each contrast and *In* ≅ 37. While the information was balanced across the 3 sets of SNPs, when one considers these two measures across all 1300 SNPs for each contrast, they also represent a balanced design (Table 2).

**Table 2:** Measures for balancing information (standard deviation) in the three information contrasts

	Information contrast		
Information	|P_EU_-P_AI_|	|P_EU_-P_AF_|	|P_AI_-P_AF_|
Number of SNPs	*N* = 450	*N* = 450	*N* = 400
Information-for-Assignment, *In*	37.3	37.3	36.7
Mean δ	0.529 (0.022) *N* = 450	0.528 (0.022) *N* = 450	0.542 (0.027) *N* = 400
All SNPs *N* = 1300			
Information-for-Assignment, *In*	56.5	56.9	58.3
Mean δ	0.351 (0.132)	0.364 (0.121)	0.350 (0.130)

The statistic *F*_*st*_ was also calculated for each set of SNPs, two ancestral populations at a time, as well as for all SNPs, two ancestral populations at a time, for each information contrast (Table 3). While the information was balanced for mean δ and *In*, the mean *F*_*st*_ were variable across contrasts.

**Table 3:** Mean *F*_*st*_ (standard deviation) in individual and combined contrasts

	Information contrast		
	|P_EU_-P_AI_|	|P_EU_-P_AF_|	|P_AI_-P_AF_|
*F*_*st*_ by contrast	0.516 (0.023) *N* = 450	0.381 (0.015) *N* = 450	0.437 (0.035) *N* = 400
*F*_*st*_ over all contrasts *N* = 1300	0.502 (0.036)	0.367 (0.018)	0.421 (0.028)

**Fig. 2:**
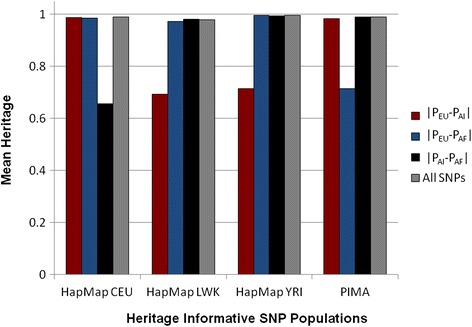
Mean ancestry when estimated with three sets of SNPs, each set maximized for information in one contrast. Each of the ancestral populations was modeled by samples from HapMap or from the Pima Indian GWAS. Three sets of SNPs were each maximized for information in one of the three contrasts and then used to estimate the respective mean ancestry (CEU, European (EU); LWK and YRI, African (AF); Pima, American Indian (AI)) in each sample, with the expectation of a mean of 1.0. When the ancestry of the sample was not represented in the maximized contrast set, then the estimates of individual ancestry become unstable with large error

Individual ancestry estimates were first computed for the 4 ancestral populations to test the validity of the method and the stability of the estimates of individual ancestry in the maximum likelihood model (Table 4, Fig. 2). The expectation was that the vector of informative SNPs would return a mean value of 1.0 for the heritage of the respective ancestry group. For the CEPH sample the mean value for European ancestry is 0.998 for the |P_EU_-P_AI_| set of SNPs, 0.986 for the contrast |P_EU_-P_AF_|, and 0.990 when estimated with all 1300 SNPs, with the AI and AF ancestry estimates being close to 0.0. However, when EU ancestry is estimated in the HapMap CEU sample using only the 400 SNPs maximized for the |P_AI_-P_AF_| contrast, then *Ex(EU) = 1.0* is not met and the mean estimates become unstable: EU = 0.656, AI = 0.185, and AF = 0.159. The same pattern holds for AF estimates in HapMap LWK and YRI and for AI estimates in the Pima; when the ancestral component is not part of the maximized contrast, then the maximum likelihood model becomes unstable and returns unreliable, incorrect estimates for individual ancestry. When the 3 sets of maximized markers are pooled, however, the model returns stable, correct estimates (Table 4, Fig. 2).

**Table 4:** Mean (standard deviation) of source samples for AIMs typed with the 3 sets of informative markers

SNPs in estimates	Source samples for AIMs											
	HapMap CEU, *N* = 165			HapMap LWK, *N* = 110			HapMap YRI, *N* = 193			Pima, *N* = 964		
	EU	AI	AF	EU	AI	AF	EU	AI	AF	EU	AI	AF
|P_EU_-P_AI_| *N* = 450	.988 (.022)	.004 (.010)	.008 (.021)	.163 (.182)	.144 (.180)	.693 (.359)	.133 (.175)	.152 (.178)	.715 (.349)	.004 (.029)	.985 (.065)	.011 (.053)
|P_EU_-P_AF_| *N* = 450	.986 (.023)	.010 (.022)	.004 (.010)	.010 (.015)	.017 (.030)	.972 (.028)	.001 (.003)	.004 (.012)	.995 (.012)	.143 (.169)	.715 (.325)	.142 (.164)
|P_AI_-P_AF_| *N* = 400	.656 (.403)	.185 (.221)	.159 (.186)	.012 (.022)	.008 (.015)	.980 (.023)	.004 (.016)	.002 (.006)	.994 (.017)	.009 (.049)	.988 (.055)	.003 (.017)
All SNPs *N* = 1300	.990 (.014)	.005 (.010)	.005 (.010)	.015 (.015)	.007 (.011	.978 (.016)	.001 (.004)	.002 (.007)	.996 (.008)	.007 (.039)	.989 (.048)	.003 (.021)

Each set is maximized for information in one contrast, and with all combined SNPs

*EU* European ancestry, *AI* American Indian ancestry, *AF* African ancestry

The above analysis was repeated for the 4 ancestral samples using the STRUCTURE Bayesian cluster method with 3 ancestral components, EU, AI, and AF (K = 3) and gave very similar results to those presented in Table 4 and Fig. 2 (Additional file 4: Table S1, Additional file 5: Figures S1–S4). In two instances for the Pima Indians, for maximized contrasts |P_AI_-P_AF_| and |P_EU_-P_AI_|, the Bayesian method did not return the expected value of AI even when the information in the contrast was maximized for this ancestral component. When the 1300 SNPs with balanced information were incorporated into the STRUCTURE program, it returned the expected mean values and proportions of ancestry in the four ancestral samples (Additional file 5: Figures S1 and S5).

**Fig. 3:**
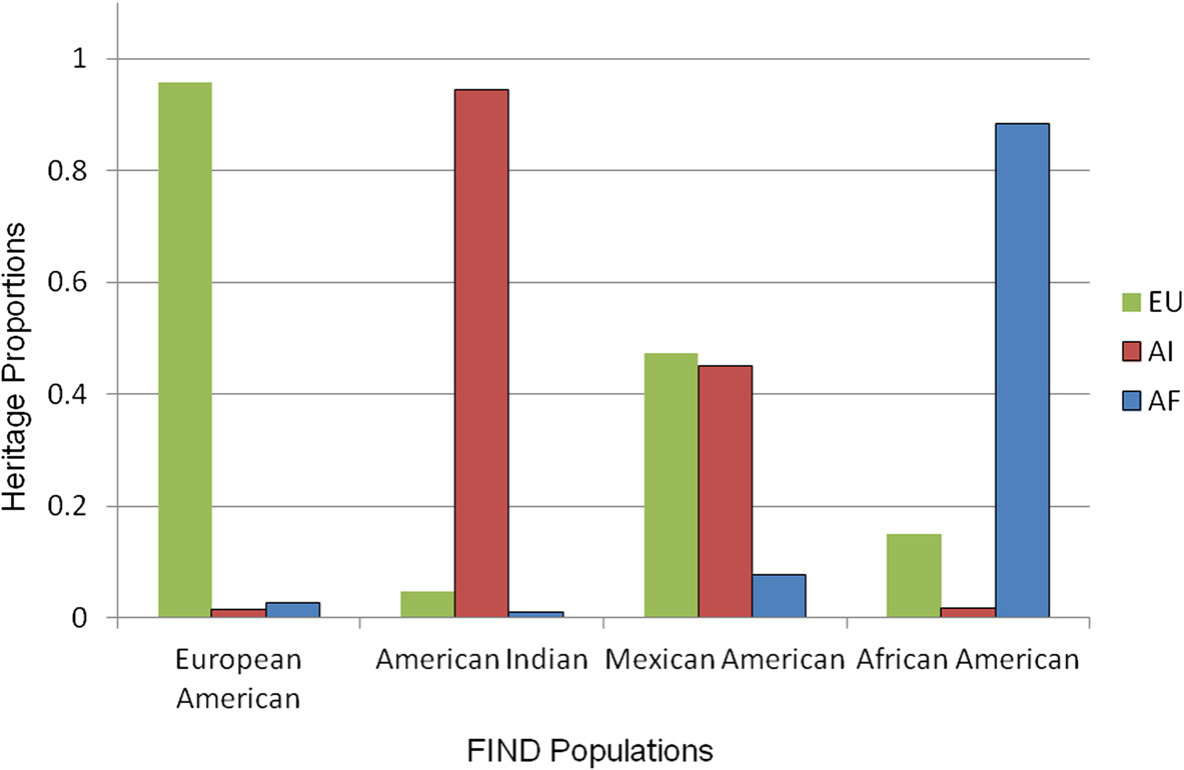
Mean heritage for persons who self-identify in the FIND study. Legend: Mean estimates are presented for the three components of individual ancestry in the FIND samples. For European Americans, American Indians, and African Americans the expected largest component is >0.8, while for Mexican Americans the European and American Indian components are similar. EU: European Ancestry; AI: American Indian Ancestry; AF: African Ancestry

The maximum likelihood individual ancestry algorithm was then applied to the four FIND samples using the pooled set of 1300 SNPs (Table 5, Fig. 3). For the FIND European Americans the EU component had a mean of 0.961 (mean standard error for individual ancestry, 0.008), with small mean proportions for AI and AF. American Indians in the FIND had a large AI mean estimate, 0.945 (0.007), with small components for EU and AF. The primary heritage in the FIND African Americans is AF, 0.830 (0.008), with the balance being primarily from European heritage, 0.149 (0.009). The FIND Mexican Americans represent their complex origin from the three heritage groups: EU 0.476 (0.012), AI 0.447 (0.011), and AF 0.077 (0.011).

**Table 5:** Mean (standard deviation) and range for individual heritage and standard error estimates for FIND populations

		EU				AI				AF			
		Heritage		Standard error		Heritage		Standard error		Heritage		Standard error	
FIND population	N	Mean	Range	Mean	Range	Mean	Range	Mean	Range	Mean	Range	Mean	Range
European American	826	.961 (.090)	.059–1.0	.008 (.002)	.002–.014	0.014 (.023)	0–.237	.011 (.002)	.003–.015	0.025 (.084)	0–.923	.008 (.002)	.001–.014
American Indian	869	.045 (.090)	0–.712	.009 (.003)	.002–.017	0.945 (.111)	0–1.0	.007 (.003)	.001–.016	0.010 (.049)	0–.866	.007 (.002)	.001–.015
Mexican American	1451	.476 (.134)	0–.974	.012 (.001)	.006–.015	0.447 (.140)	0–1.0	.011 (.001)	.004–.015	0.077 (.053)	0–.845	.011 (.001)	.005–.014
African American	1385	.149 (.104)	0–.638	.009 (.002)	.002–.016	0.021 (.030)	0–.539	.010 (.002)	.002–.016	0.830 (.111)	.058–1.0	.008 (.002)	.001–.014

IGA estimates were computed with 1300 Ancestry Informative Markers and a 3 Ancestral components Model

*EU* European heritage, *AI* American Indian heritage, *AF* African heritage

**Fig. 4:**
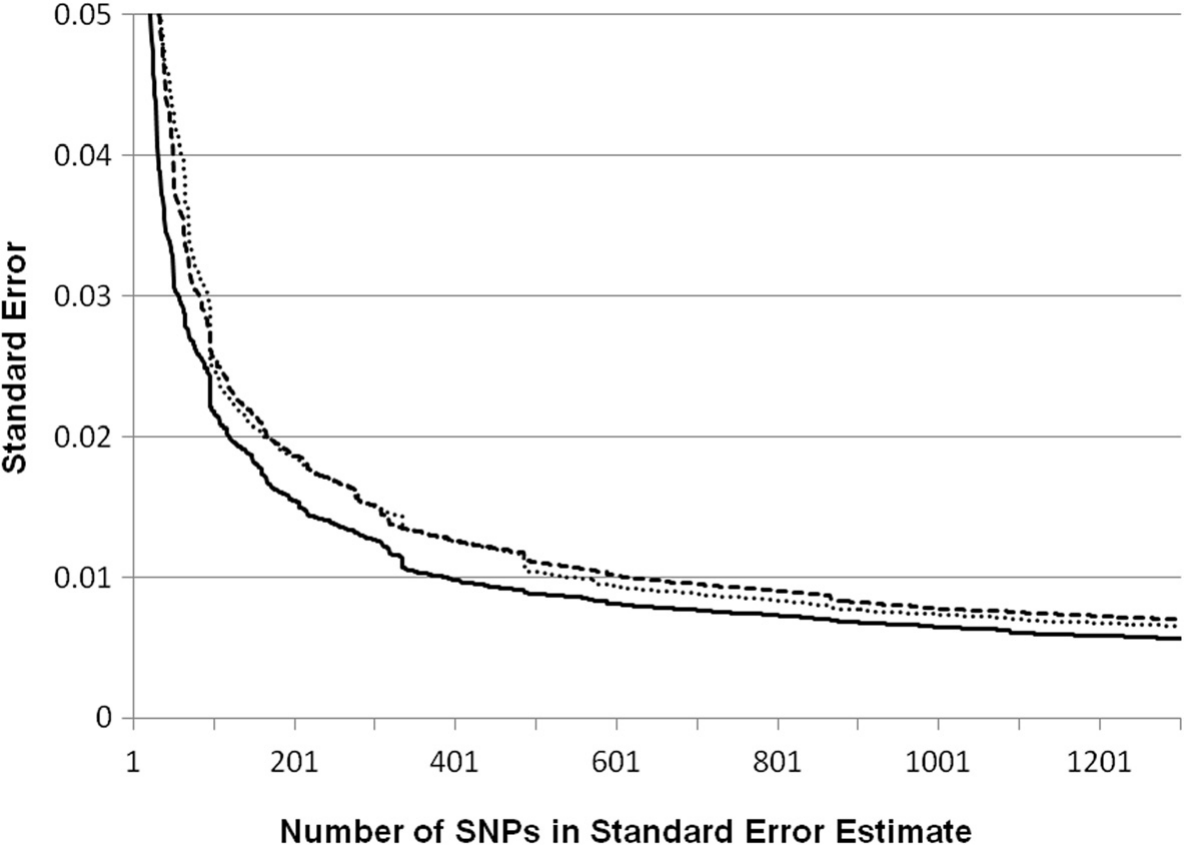
Mean standard error of individual heritage estimates in four FIND samples by number of SNP Loci. The mean standard error of the individual ancestry estimates was calculated across the 4 FIND samples at 1300 points, adding each successive SNP to the calculation in chromosome and position order (EU, dotted line; AI, dashed line; AF, solid line). After the addition of about 200 informative SNPs, the standard error falls below 0.02 and decreases further at a slower rate with each additional locus. It takes approximately 700 SNPs in the estimates to have a mean standard error <0.01

The standard error for each individual ancestry estimate was calculated from the *2x2* information matrix for each person over the vector of non-missing SNPs for the estimate. Figure 4 illustrates the effect on the standard error of adding SNPs to the estimate. The cumulative standard error was calculated for SNPs 1 to 1300, in chromosome and position order, and then averaged at each point over the four FIND populations for the EU, AI, and AF ancestral components. To insure a mean standard error <0.01, approximately 700 SNPs are necessary for the maximum likelihood model.

**Fig. 5:**
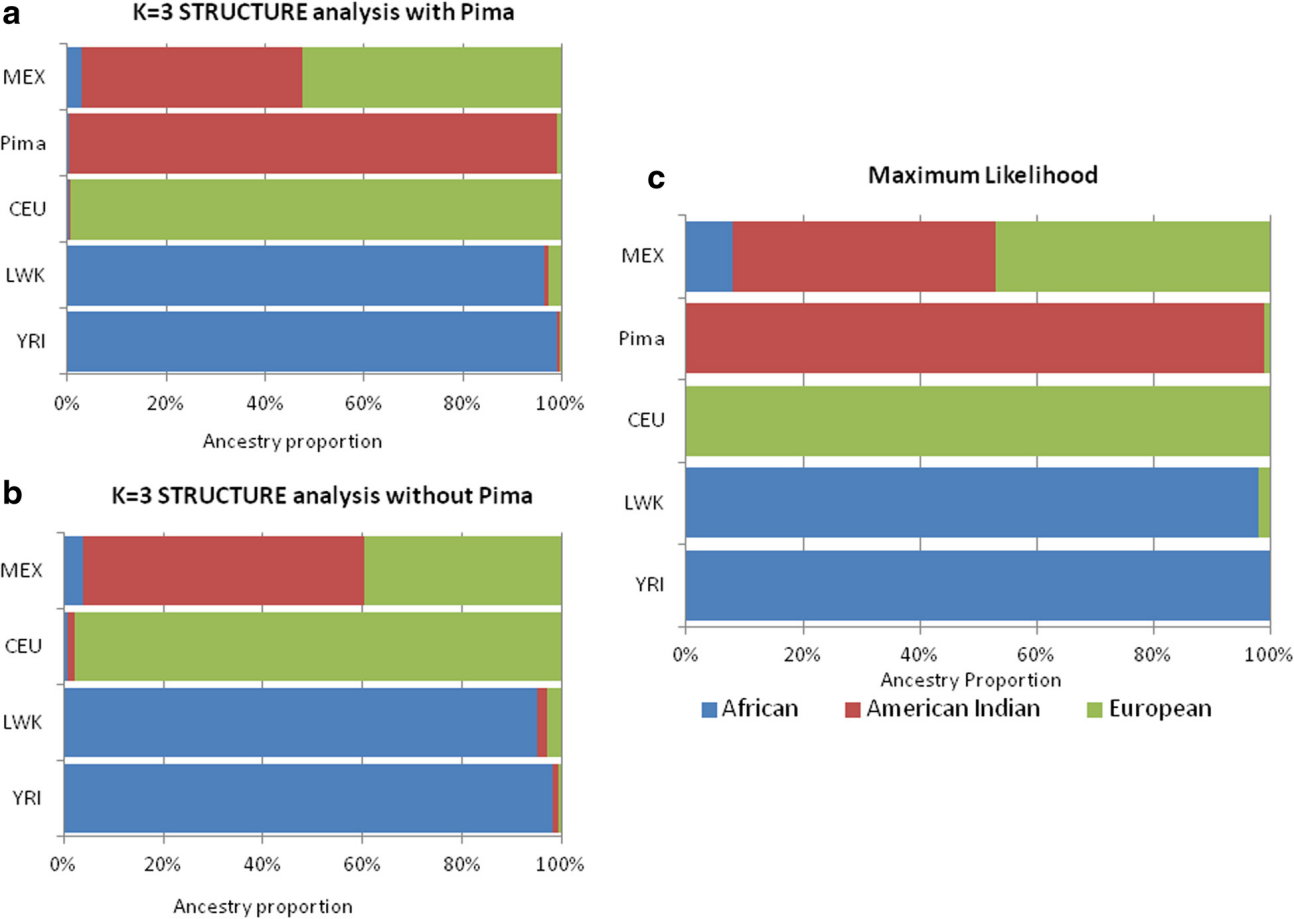
Estimates of individual heritage for the FIND Mexican American sample with and without the Pima genotypes. Panel **a** has the estimates from STRUCTURE while using the 1300 genotypes from the Pima, CEU, LWK, and YRI samples. These are very similar to the estimates obtained from the maximum likelihood method that is presented in Panel **c**. When the Pima genotypes were removed from the STRUCTURE analysis, the amount of American Indian ancestry was overestimated in the Mexican sample in Panel **b**. It is recommend that, in the latter situation, maximum likelihood returns the better estimates of individual heritage

The performance of these markers in an admixed population using the Bayesian method was assessed by the STRUCTURE program for the FIND Mexican Americans with 3 ancestry components (K = 3). When genotypic data representative of the three ancestral reference populations were included (CEU, YRI + LWK, Pima Indians), the overall admixture proportions were very similar to those obtained with the maximum likelihood method (Fig. 5, Panel a). Since individual level data may not be readily available for a suitable American Indian reference population, the analyses were repeated without including the Pima data as a reference. In this situation, the Amerindian component in the FIND Mexican American participants was modestly overestimated in comparison to the case when the Pimas were included, while the European component was underestimated (Fig. 5, Panel b).

An alternate method for controlling for population structure in GWAS is to calculate the PCs from the samples. To compare the PC and heritage estimates, a Pearson correlation coefficient was calculated for the 3 admixture components and the first 2 PCs for the combined sample (*N* = 4391). The EU heritage component was highly correlated with PC2 [0.9954 (95 % C.I. 0.9951, 0.9957)], while the AF heritage had a more modest correlation [0.9111 (0.9060, 0.9160)] with PC1. American Indian heritage was negatively correlated with both PC1 and PC2 [−0.8600 (−0.8675, −0.8521) and −0.5067 (−0.5283, −0.4843)]. When the three admixture components were each used as a dependent variable in a linear regression with PC1 and PC2 as explanatory variables, the R-square values were close to 1.0: EU (0.995), AI (0.996), and AF (0.996).

To assess the potential role of ancestry in confounding associations with diabetic nephropathy, each of the three heritage estimates was first tested singly for association, with the covariates, for each of the 4 FIND populations (Additional file 4: Tables S2, S3 and S4). While the variables enrolled age, sex, and enrolment center were consistently associated with the disease, there was no significant association with any individual heritage variable when tested within each sample. However, when the samples were combined (*N* = 4126) it introduced population structure and each heritage variable had a significant odds ratio when tested singly in the model: EU odds ratio 0.338, *p* < 0.0001; AI 1.960, 0.028; and AF 2.519, *p* < 0.0001;

Logistic regressions were repeated in the combined sample with the same covariates and two individual heritage variables at a time (Table 6, Additional file 4: Tables S3 and S4). Given that the heritage values for each person sum to 1.0, the variable that is left out of the logistic model is the reference for the other two in the computation of the odds ratio. When EU and AI are included, with AF as a reference, EU is significantly less than 1.0 while the 95 % confidence contrast of AI includes 1.0. When EU and AF are in the same model, with AI as a reference, a similar pattern results. Finally, when EU is the reference for AI and AF, both covariates have odds ratios greater than 1.0: AI 3.762, *p* < 0.0001 and AF 2.956, *p* < 0.0001.

**Table 6:** Tests for the association of heritage with diabetic nephropathy in the combined FIND populations, *N* = 4126

	EU heritage	p	AI heritage	p	AF heritage	p
Model 1	0.311 (.232, .418)	<.0001	1.031 (.547, 1.944)	0.924	Reference	
Model 2	0.269 (.143, .507)	<.0001	Reference		0.748 (.381, 1.468)	0.398
Model 3	Reference		3.762 (1.958, 7.228)	<.0001	2.956 (2.212, 3.947)	<.0001

Logistic models have two heritage variables in addition to explanatory variables Enrolled-Age, Sex, and Enrolment Center. Results are presented as Odds Ratios (95 % C.I.). (For covariate results see Additional file 4: Tables S3 and S4.)

## Discussion

### A panel of SNPs informative for African, American Indian and European ancestry

A panel of 1300 SNPs was developed which can serve as informative markers for African, American Indian and European ancestry; these ancestry components are often of interest in genetic epidemiologic studies of populations from the Americas. Although other similar marker panels have been developed, the samples used as the American Indian ancestral group were few and potentially admixed. The present panel was developed using a large sample of American Indians; although the samples derived from a single tribe, the Pima Indians of Arizona, there is minimal European admixture in this population [10]. The SNPs are useful for estimation of global ancestry across the genome. Estimation of local ancestry at specific genomic regions requires more dense genotypic data, which may not be available to all investigators. Although local ancestry estimates can be useful for mapping studies, when they are used as covariates it can result in over-adjustment, whereas adjustment for global ancestry is more useful to reduce confounding in GWAS [19–21]. Further, the association of global ancestry with disease risk may be of interest in itself in some genetic epidemiologic applications. Thus, the present set of SNPs, or a subset of them, may be useful for genetic epidemiologic studies. If a subset of the markers is chosen, it is important to balance the information regarding the contrasts among ancestral populations.

### An information contrast will only return reliable estimates for two ancestral populations

When a model for individual ancestry estimates has only two ancestral populations in the AIMs set, then the balance of the model is not in question because there is only one allele frequency contrast for each AIM, |P_1_-P_2_|. However, when a poly-ancestry model (>2) is created, then all allele frequency difference contrasts must be considered. For a model with 3 ancestral populations (Fig. 1) the contrasts |P_1_-P_2_|, |P_1_-P_3_|, and |P_2_-P_3_| must be integrated into the estimates. But, as we have shown, each contrast is still only reliably informative for the two ancestral populations in it.

We chose to demonstrate this with the extreme case by choosing 3 sets of AIMs that were each maximized for information in only one contrast (δ ≥ 0.5 in the chosen contrast and δ < 0.3 in the other two) and then using each set to estimate all of the ancestral components (Table 4). When the ancestry is for one of the two ancestral populations in the contrast, then the maximum likelihood model is balanced and provides accurate estimates. When one tries to estimate an ancestral component for which the markers contained in the maximized contrast set are not informative, then the model is unbalanced and the estimates are not correct. Also, the error in the unbalanced design appears to be random and distributed equally in the two ancestral components that are not part of the ancestral sample. In addition, if no standard error of the estimate is computed, there are no internal signals that would indicate that the estimates are incorrect. When the information is unbalanced, the internal signal for incorrect estimates is a large standard error. Even with the unbalanced design, the computer algorithm maximizes the likelihood and provides 3 estimates of ancestry for each person. This fact highlights the need to validate each set of SNPs that is incorporated into a maximum likelihood model for ancestry by testing them with the individuals in the ancestral populations from which the AIMs were chosen: the expected mean value should be 1.0 for the respective ancestral component.

### Accurate ancestry estimates require careful balancing of information between contrasts

To insure the accuracy of the ancestry estimates the information in the 3 contrasts of the 3-ancestry model must be balanced (Table 2). There are many approaches possible to address this problem The key is to balance the information over all SNPs for the three contrasts, whether or not a single AIM is informative for either one or two contrasts. This becomes more difficult with two-contrast informative SNPs because, when trying to balance the model, each addition or subtraction affects two information statistics. One strategy, shown in the present work, is to choose three sets of single contrast informative SNPs. A second approach is to choose a set of double informative SNPs, such as ones with |P_1_-P_2_| and |P_1_-P_3_| informative, and balance these with single informative |P_2_-P_3_| loci.

### Using a Bayesian clustering method with K = 3 does not obviate the need for balanced information in the ancestry markers

Repeating the individual admixture estimates using the STRUCTURE program (K = 3) gave similar results to the fixed parental allele frequency algorithm but showed, in addition, that it was even more sensitive to imbalances in information. It did not return the expected mean value of AI for Pima Indians even when the contrast, |P_AI_-P_AF_| or |P_EU_-P_AI_|, was maximized for this component (Additional file 4: Table S1); whereas the fixed parental allele algorithm always returned the correct mean expected values for the components maximized in the contrast when all 3 components were being simultaneously estimated (Table 4, Fig. 2). When the Bayesian cluster algorithm was used with all 1300 SNPs with balanced information, it returned the appropriate mean expected values for all ancestry samples. This further illustrates the need for careful balancing of the ancestral information when selecting markers, irrespective of whether the algorithm uses a classical method such as maximum likelihood or a more recent method such as STRUCTURE.

Previous studies have shown that, given sufficient information, maximum likelihood methods, Bayesian methods such as STRUCTURE and hybrid methods produce similar admixture estimates [22, 23]. For optimal ancestry estimates, all methods require information on allele frequencies in the ancestral populations, either by taking them as known quantities, as in the classic maximum likelihood method used here, or by inclusion of genotypes from representative ancestral reference groups as in STRUCTURE [22, 23]. Raw genotypic data from a suitable American Indian reference ancestry population may not be readily available, however, and in the absence of these data there was a modest overestimation of the Amerindian component in the FIND Mexican Americans when STRUCTURE was used (Fig. 5, Panel b). In the absence of genotypic data from an American Indian reference ancestry group, the maximum likelihood method with specified ancestral allele frequencies is preferable (Fig. 5, Panel c). Given genotypes on some of the AIMS, this method can be readily implemented with the allele frequencies provided in supplementary tables of American Indian (Pima) SNP allele frequencies used in the present study.

### Balancing information in contrasts minimizes the error in replicate tests

A second set of 975 AIMs (Additional file 6) was chosen to investigate the error when individual heritage is estimated in the same person with two balanced sets of SNPs. It was also applied to the four ancestral populations in the present study and the distribution of the heritage differences was examined. For the HapMap CEU sample the mean difference for EU heritage was −0.003 with a median and mode value of 0.000 with the distribution of the differences being relatively symmetrical on either side of the mean (Additional file 4: Table S5). Very similar results were obtained for the distributions of AF heritage in the HapMap LWK and YRI samples and for AI heritage in the Pima. Therefore balancing information in the contrasts of the AIMs creates “correct” estimates of individual heritage by minimizing error inherent in the algorithm and the vector of AIMs, and emphasizes the importance of including the standard error or 95 % confidence contrasts with any point estimate of individual genetic heritage.

### The FIND samples

The distribution of mean IGA in the FIND samples represents the creation of new American populations from immigrants from historically separated parental groups. African Americans in the FIND have 83.3 % of their genome derived from Africa and about 15.1 % from Europe, while there is only a small component from American Indians (Table 5). The genetic composition of African American populations can vary greatly by geographical location, whether urban or rural, north or south. Parra et al. [24] estimated EU by weighted least squares (WLS) in 10 urban African American samples and reported proportions from 0.116 (Charleston, S.C.) to 0.225 (New Orleans). An isolated population, the Gullah Sea Islanders off the coast of South Carolina, had an EU contribution of only 0.035 [25]. A more recent estimate of IGA in 228 African Americans recruited by the University of Connecticut Health Center reported: EU, 0.17; AF, 0.75; and AI, 0.08 [26]. Therefore the proportion of EU-derived genes in the FIND AA sample accords well with reports for urban African Americans in the United States.

Persons who self-identify as Mexican Americans in the southwest United States have reported admixture that is consistent from California to Texas. Long et al., in 730 unrelated persons from paternity tests in Arizona, reported WLS proportions EU 0.68, AI 0.29, and AF 0.03 and that these proportions are within one standard error of the mean from proportions reported from San Antonio, Texas, and Los Angeles, California [27]. The Arizona sample was later enlarged to 2249 persons with revised WLS proportions EU 0.616, AI 0.314, and AF 0.071 and correspondingly smaller standard errors. Additional Mexican American admixture proportions (EU, AI and AF, respectively) have been reported from the San Antonio Diabetes Study (0.502, 0.464, 0.031) and the San Antonio center for Biomarkers of Risk of Prostate Cancer (0.589, 0.382, 0.029) [28]. In two case–control studies of breast cancer in Latinas in the San Francisco Bay area, genetic admixture was measured; Fejerman et al. reported proportions EU 0.53, AI 0.40, AF 0.07 in 597 controls and 0.58, 0.35, 0.07 in 440 cases in women born in the U.S. [29]; Ziv et al. stratified their sample by 175 women born in Mexico, EU 0.520, AI 0.443, AF 0.037, and 100 persons born in the U.S. whose grandparents were Mexican-born, 0.473, 0.478, 0.048 [30]. The FIND Mexican American proportions (Table 5) fit well within these and other data reported in the literature, that the European American component is the largest in the range of 0.45-0.65 followed by a smaller American Indian component and 0.03–0.07 African admixture. As the sample size increases, and the number of American Indian informative SNPs becomes larger in the estimate, the fraction of European admixture appears to decrease while that of American Indians increases.

While variation across studies appears to be the norm, the variation within the FIND Mexican American sample is relatively consistent when stratified by sex and enrolment center. The 554 males (EU 0.482, AI 0.446, AF 0.072) and the 846 females (EU 0.467, AI 0.456, AF 0.077) are well within one standard deviation for all three proportions. When the 4 enrolment centers that have sample sizes greater than 25 are considered (center 2, *N* = 634; 3, 114; 4, 308; and 5, 318), the range of proportions is small: EU 0.456–0.486, AI 0.443–0.482, and AF 0.071–0.076. Centers 2, 3, and 5 are in California, while center 4 is in Texas. Therefore, the FIND Mexican Americans, when IGA is estimated with the 1300 informative markers, exhibit a relatively uniform distribution of admixture across a large geographical area.

In contrast with FIND African American and Mexican American samples, the European American and American Indian samples exhibit small amounts of genetic admixture (Table 5). Persons who self-identify as of European heritage have only 1.5 % AI and 2.6 % AF mean heritage. Full Heritage Pima Indians make up a large proportion of the 869 American Indians who were recruited for the FIND; the amount and origin of their genetic admixture has been reported [10, 13, 31]. Pima Indians lie on the western end of a cline of European admixture that has its highest values in the northeastern United States, falls into intermediate levels in the Midwestern states, and reaches its lowest level in the desert southwest. This cline generally comports with the settlement of the country by persons of European origin from east to west. European IGA in the Pima Indians can be traced primarily to their genetic and cultural relations with the people of Mexico since the Spanish first entered the new world [10]. The IGA estimates derived by the present method, and most other commonly used methods, assume Hardy-Weinberg equilibrium, and this assumption may not hold in some situations, such as a case–control study when markers are associated with disease; however, simulation studies have shown that admixture estimates are generally robust to deviations from Hardy-Weinberg equilibrium [32].

### Standard error of the estimate

An advantage of the maximum likelihood method for individual ancestry estimation is the ability to calculate the information matrix and invert it for estimates of the variances, because point estimates of population parameters have little meaning without a measure of error accompanying them. Figure 4 illustrates that the standard error of individual ancestry has its largest improvement, decrease, within the first 100 informative SNPs in the estimates. After this there is steady improvement in the precision of the numbers, though the average effect of each additional AIM becomes progressively less. However, increasing the number of SNPs can have a significant effect on the confidence intervals of the individual heritage estimates. Gaining this additional precision could be important when the magnitude of estimated ancestry is small. At approximately 700 SNPs the mean standard errors are below 0.01, while with 1300 AIMs in the estimate the average standard error is in the range of 0.006–0.008.

### Maximum likelihood ancestry estimates versus principle components for measuring population structure

In the FIND samples, the principal components derived from the GWAS SNPs and the ancestry estimates derived from the AIMs capture largely the same information, but, as they represent somewhat different functions of the data, the interpretation of the variables may differ. The relative advantage of PCs to account for population structure in association studies, compared to heritage estimates, is their relative ease of calculation and they do not require an a priori specification of ancestral populations. However their primary disadvantage is the ambiguity of their biological meaning. Maximum likelihood individual ancestry estimates, with standard errors, have the advantage of a clear biological meaning. Each proportion represents the fraction of alleles in the individual’s genome from an historical ancestral population. The disadvantage of the maximum likelihood method as currently implemented is the need for a large set of parental frequencies that are unlinked, balanced in their information, and with low replicate error rates in the SNP genotyping. The computational burden of maximum likelihood is also higher than for PCs. If these conditions can be met, however, heritage estimates can have great utility for tests of admixture equilibrium, monitoring information, and computing odds ratios as a function of individual heritage, as well as being used as covariates in tests of association in GWAS.

### Population structure from combining samples leads to the association of ancestry and diabetic nephropathy

Tests of the association of diabetic nephropathy and IGH were computed separately for each of the 4 FIND samples in a logistic regression with enrolled age, sex, and enrollment center as covariates (Additional file 4: Tables S2, S3 and S4); no IGH component had a statistically significant association with disease in the individual samples. When the three tests were performed in the combined sample all IGH components were associated with diabetic nephropathy. To further parse the associations, a second set of logistic regressions was performed on the combined sample while assessing two heritage components at a time and using the third heritage as a reference with sex, enrolled age, and enrollment center again as covariates (Table 6). With AF heritage as reference, persons of European heritage are protected from the disease, while persons with AI heritage do not have an odds ratio statistically different from 1.0, which suggests that their odds ratio is similar to those with African heritage. A symmetrical result occurs when AI heritage is the reference; EU heritage is again protective while the odds ratio for AF is not statistically different from 1.0. This is confirmed further by the model that tests AI and AF heritage with EU as reference, in which both AI heritage and AF heritage are significantly greater than 1.0 while their 95 % confidence intervals overlap. While these estimates cannot necessarily be interpreted as reflective of population risk because of the way that patients are recruited in FIND, the odds ratios resulting from the population structure of the combined sample do generally reflect what is known about the relative occurrence and risk of diabetic nephropathy in the 4 heritage groups.

## Conclusions

1. Failure to balance AIM information in poly-ancestry models creates biased estimates of individual admixture with large error. This occurs whether one employs the fixed parental allele algorithm for estimating IGA or the Bayesian clustering method as implemented in the program STRUCTURE. It is very important to describe the information contrasts explicitly and then emphasize the attention to them that is needed to compute correct estimates with low error because many researchers who are not trained in the details of the algorithms are downloading code, choosing sets of AIMs, and applying these to their analysis of population structure.
2. A set of ancestry informative markers is provided for estimating American Indian ancestry that reflects an ancestral tribe from the Paleo-Indian migration across the Bering Strait, the Pima Indians [33], who are the most completely characterized Indian group in North America. These AIMs will be particularly useful for estimating genetic admixture in populations from the Americas.
3. A statistic with no measure of error has very limited meaning and utility. Our method provides the researcher with a tool to construct 95 % confidence intervals for IGA and to gage how many SNPs are necessary to achieve a desired mean error in the sample.
4. We parse population structure by estimating both IGA and PCs and show that the two methods are highly correlated and useful for adjusting for structure in association studies, and suggest that IGA has the further advantage of being a number that is more easily understood in the context of the sample than are PCs.
5. We test the association of IGA with diabetic nephropathy in the FIND in both the individual and combined samples and demonstrate how combining samples to increase power in a genome wide association study can create associations between ancestry and the disease. We then find that the odds ratios for the associations of IGA with disease in the combined sample are consistent with what is known about the incidence and prevalence of diabetic nephropathy in these populations. Therefore we exploit population structure to provide us with useful information about the relative occurrence of the disease among the groups.

### Data availability

All FIND phenotype and genotype files, except those for the American Indian subjects, are available from the dbGAP database (accession number phs000333.v1.p1). Data for the American Indian subjects are not publically available for privacy reasons. Interested researchers who meet the criteria for access to the data can contact: Robert Hanson (rhanson@phx.niddk.nih.gov) or Clifton Bogardus (cbogardus@phx.niddk.nih.gov).

### Ethics approval and consent to participate

The FIND was completed in accordance with the principles of the Declaration of Helsinki. Written informed consent was obtained from all participants. The Institutional Review Board at each participating center (Case Western Reserve, Cleveland, Ohio; Harbor University of California Los Angeles Medical Center; Johns Hopkins University, Baltimore; National Institute of Diabetes and Digestive and Kidney Diseases; University of California, Los Angeles, CA; University of New Mexico, Albuquerque, NM; University of Texas Health Science Center at San Antonio, San Antonio, TX; Wake Forest School of Medicine, Winston-Salem, NC) approved all procedures, and all study subjects provided written informed consent. A certificate of confidentiality was filed at the National Institutes of Health.

### Consent for publication

Publication of the results of the analyses was part of the informed consent. No individual-level clinical data were published.

## Appendix

In order to calculate the variance of the ancestral components the information matrix must first be computed as

$$ \left[\begin{array}{cc}\hfill {\displaystyle \sum_{g=1}^G}{I}_g^{m_1}\hfill & \hfill {\displaystyle \sum_{g=1}^G}{I}_g^{m_1{m}_2}\hfill \\ {}\hfill {\displaystyle \sum_{g=1}^G}{I}_g^{m_1{m}_2}\hfill & \hfill {\displaystyle \sum_{g=1}^G}{I}_g^{m_2}\hfill \end{array}\right] $$

where the information elements are derived from the second derivative of the likelihood function. First the hybrid frequencies for the *g*^*th*^ SNP are calculated with the maximum likelihood values of the individual admixture proportions *mlm*_*1*_ and *mlm*_*2*_

$$ {P}_{hA1g} = {p}_{31g} + ml{m}_1\varDelta {1}_g + ml{m}_2\varDelta {2}_g $$

$$ {P}_{hA2g} = {p}_{32g} + ml{m}_1\varDelta {3}_g + ml{m}_2\varDelta {4}_g. $$

Each of the *G* genotypes produces an element for the three distinct summations in the information matrix. For genotype *A1A1*, the three contributions to the matrix can be computed as:

$$ {I}_g^{m_1}={\left(2\varDelta {1}_g/\left({P}_{hA1g}\right)\right)}^2/{\left({P}_{hA1g}\right)}^2 $$

$$ {I}_g^{m_1{m}_2}=\left(\frac{\left(4\varDelta {1}_g\varDelta {2}_g\right)}{{\left({P}_{hA1g}\right)}^2}\right)/{\left({P}_{hA1g}\right)}^2 $$

$$ {I}_g^{m_2}={\left(2\varDelta {2}_g/\left({P}_{hA1g}\right)\right)}^2/{\left({P}_{hA1g}\right)}^2. $$

For the heterozygote *A1A2* the respective contributions are:

$$ {I}_g^{m_1}={\left(\frac{\varDelta {1}_g}{P_{hA1g}}+\frac{\varDelta {2}_g}{P_{hA2g}}\right)}^2/\left(2{P}_{hA1g}{P}_{hA2g}\right) $$

$$ {I}_g^{m_1{m}_2}=\left(\frac{\varDelta {1}_g}{P_{hA1g}}+\frac{\varDelta {3}_g}{P_{hA2g}}\right)\left(\frac{\varDelta {2}_g}{P_{hA1g}}+\frac{\varDelta {4}_g}{P_{hA2g}}\right)/\left(2{P}_{hA1g}{P}_{hA2g}\right) $$

$$ {I}_g^{m_2}={\left(\frac{\varDelta {2}_g}{P_{hA1g}}+\frac{\varDelta {4}_g}{P_{hA2g}}\right)}^2/\ \left(2{P}_{hA1g}{P}_{hA2g}\right). $$

And for *A2A2*:

$$ {I}_g^{m_1}={\left(2\varDelta {3}_g/\left({P}_{hA2g}\right)\right)}^2/{\left({P}_{hA2g}\right)}^2 $$

$$ {I}_g^{m_1{m}_2}=\left(\frac{\left(4\varDelta {3}_g\varDelta {4}_g\right)}{{\left({P}_{hA2g}\right)}^2}\right)/{\left({P}_{hA2g}\right)}^2 $$

$$ {I}_g^{m_2}={\left(2\varDelta {4}_g/\left({P}_{hA2g}\right)\right)}^2/{\left({P}_{hA2g}\right)}^2. $$

The variances and covariance are calculated as

$$ {\left[\begin{array}{cc}\hfill {\displaystyle \sum_{g=1}^G}{I}_g^{m_1}\hfill & \hfill {\displaystyle \sum_{g=1}^G}{I}_g^{m_1{m}_2}\hfill \\ {}\hfill {\displaystyle \sum_{g=1}^G}{I}_g^{m_1{m}_2}\hfill & \hfill {\displaystyle \sum_{g=1}^G}{I}_g^{m_2}\hfill \end{array}\right]}^{-1}=\left[\begin{array}{cc}\hfill V\left( ml{m}_1\right)\hfill & \hfill Cov\left( ml{m}_1, ml{m}_2\right)\hfill \\ {}\hfill Cov\left( ml{m}_1, ml{m}_2\right)\hfill & \hfill V\left( ml{m}_2\right)\hfill \end{array}\right] $$

$$ V\left( ml{m}_3\right) = V\left( ml{m}_1\right) + V\left( ml{m}_2\right)+2Cov\left( ml{m}_1, ml{m}_2\right) $$

with standard error

$$ SE\left( ml{m}_i\right)=\sqrt{V\left( ml{m}_i\right)}. $$

## Additional files

Additional file 1: RCWilliamsContrastEU_AI450AIMs. (XLSX 60 kb) Additional file 2: RCWilliamsContrastEU_AF450AIMs. (XLSX 60 kb) Additional file 3: RCWilliamsContrastAI_AF400AIMS. (XLSX 55 kb) Additional file 4: Table S1. Mean of Source Samples for AIMs typed with the STRUCTURE Software Program (K = 3). **Table S2.** Tests for the Association of Heritage with Diabetic Nephropathy in the FIND Populations. **Table S3.** Beta Coefficients for Enrolment Centers in the Logistic Regressions for the FIND Study. **Table S4.** Beta Coefficients for Sex (Women) and Enrolment Age in the Logistic Regressions for the FIND Study. **Table S5.** Distribution of the Differences in Individual Genetic Heritage for Estimates from Two, Independent, Balanced Sets of SNPs: 1300 AIMs in the first and 975 AIMs in the second. (DOCX 24 kb) Additional file 5: Figure S1. Repeating the individual admixture estimates using the STRUCTURE program (K=3). **Figure S2.** STRUCTURE estimates for four ancestral populations with 400 SNPs maximized for the American Indian-African contrast. **Figure S3.** STRUCTURE estimates for four ancestral populations with 450 SNPs maximized for the European-American Indian contrast. **Figure S4.** STRUCTURE estimates for four ancestral populations with 450 SNPs maximized for the European-African contrast. **Figure S5.** STRUCTURE estimates for four ancestral populations with all 1300 SNPs with balanced information for each contrast. (DOCX 1104 kb) Additional file 6: RCWilliamsBalanced975AIMs. (XLSX 120 kb)
