# Supplementary material for: Selecting SNPs informative for African, American Indian and European Ancestry: application to the Family Investigation of Nephropathy and Diabetes (FIND)
Source: BMC Genomics. 2016 May 4;17:325. doi: 10.1186/s12864-016-2654-x (PMC4855449; doi:10.1186/s12864-016-2654-x)
Supplement: Additional file 4: Table S1. — Mean of Source Samples for AIMs typed with the STRUCTURE Software Program (K = 3). Table S2. Tests for the Association of Heritage with Diabetic Nephropathy in the FIND Populations. Table S3. Beta Coefficients for Enrolment Centers in the Logistic Regressions for the FIND Study. Table S4. Beta Coefficients for Sex (Women) and Enrolment Age in the Logistic Regressions for the FIND Study. Table S5. Distribution of the Differences in Individual Genetic Heritage for Estimates from Two, Independent, Balanced Sets of SNPs: 1300 AIMs in the first and 975 AIMs in the second. (DOCX 24 kb) [file 12864_2016_2654_MOESM4_ESM.docx]

**Supplementary Table 1 - Mean of Source Samples for AIMs typed with the STRUCTURE Software Program (K = 3).** Three sets of informative markers were used, each set maximized for information in one contrast, and then with all combined SNPs: EU, European Ancestry; AI, American Indian Ancestry; AF, African ancestry.

| SNPs in Estimates | Source Samples for AIMs | | | | | | | | | | | |
| --- | --- | --- | --- | --- | --- | --- | --- | --- | --- | --- | --- | --- |
|  | HapMap CEU, N=165 | | | HapMap LWK, N=110 | | | HapMap YRI, N=193 | | | Pima, N=964 | | |
|  | EU | AI | AF | EU | AI | AF | EU | AI | AF | EU | AI | AF |
| \|P_EU_-P_AI_\|  N=450 | .990 | .005 | .006 | .511 | .241 | .247 | .493 | .254 | .253 | .011 | .460 | .529 |
| \|P_EU_-P_AF_\|  N=450 | .993 | .005 | .002 | .009 | .018 | .973 | .002 | .003 | .996 | .032 | .941 | .027 |
| \|P_AI_-P_AF_\|  N=400 | .247 | .289 | .464 | .005 | .008 | .987 | .003 | .003 | .995 | .502 | .491 | .008 |
| All SNPs  N=1300 | .996 | .002 | .002 | .016 | .005 | .979 | .002 | .002 | .996 | .010 | .987 | .003 |

**Supplementary Table 2 -Tests for the Association of Heritage with Diabetic Nephropathy in the FIND Populations.** The logistic models were controlled for Enrolled-Age, Sex, and Enrolment Center Sampling Variance. Each Cell represents the Odds Ratio (95% C.I.) of One Logistic Regression with the single heritage variable within the respective population. (For covariate results see Supplementary Tables 3 and 4.)

| FIND  Population | N | EU  Heritage | p | AI  Heritage | p | AF  Heritage | p |
| --- | --- | --- | --- | --- | --- | --- | --- |
| European Americans | 694 | 0.363  (.060, 2.181) | 0.268 | 0.241  (0, 400.2) | 0.707 | 3.310  (.493, 22.2) | 0.218 |
| American Indians | 768 | 0.365  (.057, 2.328) | 0.286 | 2.085  (.476, 9.134) | 0.329 | 0.642  (.021, 19.3) | 0.799 |
| Mexican Americans | 1374 | 0.868  (.373, 2.020) | 0.743 | 1.359  (.596, 3.102) | 0.465 | 0.128  (.007, 2.406) | 0.169 |
| African Americans | 1290 | 0.537  (.142, 2.032) | 0.360 | 0.175  (.001, 37.9) | 0.526 | 1.912  (.547, 6.679) | 0.310 |
| Combined | 4126 | 0.338  (.257, .444) | <.0001 | 1.960  (1.076, 3.568) | 0.028 | 2.519  (1.921, 3.304) | <.0001 |

**Supplementary Table 3 - Beta Coefficients for Enrolment Centers in the Logistic Regressions for the FIND Study.** Dependent Variable is Diabetic Nephropathy. Reference Variable (Ref.), p <0.05 (*), p < 0.01 (**), p < 0.001(***), p< 0.0001 (****).

| Logistic Model | | Enrolment Center | | | | | | | | | | | | | | | |
| --- | --- | --- | --- | --- | --- | --- | --- | --- | --- | --- | --- | --- | --- | --- | --- | --- | --- |
| Find  Sample | IGH  Covariate | cwru | cwru  oof | jhu | loyola | niddk  phx | niddk  phxoof | uab | uc | uc  irvine | ucla | ucla  harb | uclaharb  off | unm | uths  csa | wake  for | wakefor  off |
| Eu. Am. | EU | .423 | -2.80  **** |  | .524 |  |  |  |  |  |  |  | .444  * |  |  | .194 | Ref. |
| Eu. Am. | AI | .433 | -2.77  **** |  | .515 |  |  |  |  |  |  |  | .456  * |  |  | .183 | Ref. |
| Eu. Am. | AF | .422 | -2.80  **** |  | .525 |  |  |  |  |  |  |  | .451  * |  |  | .192 | Ref. |
| Mex. Am. | EU |  |  |  |  |  |  |  |  | .814  *** | 1.18  **** | .119 |  |  | Ref. |  |  |
| Mex. Am. | AI |  |  |  |  |  |  |  |  | .807  *** | 1.18  **** | .117 |  |  | Ref. |  |  |
| Mex. Am. | AF |  |  |  |  |  |  |  |  | .822  *** | 1.20  **** | .131 |  |  | Ref. |  |  |
| Am. Ind. | EU |  |  |  |  | -.035 | -2.38  **** |  |  |  |  |  |  | Ref. |  |  |  |
| Am. Ind. | AI |  |  |  |  | -.012 | -2.35  **** |  |  |  |  |  |  | Ref. |  |  |  |
| Am. Ind. | AF |  |  |  |  | .051 | -2.29  **** |  |  |  |  |  |  | Ref. |  |  |  |
| Af. Am. | EU | -.810  *** | -4.23  **** | .761  ** | -1.51  ** |  |  | .264 | -.993 |  |  | -2.32  **** |  |  |  | -1.27  **** | Ref. |
| Af. Am. | AI | -.830  *** | -4.24  **** | .748  ** | -1.51  ** |  |  | .271 | -.982 |  |  | -2.31  **** |  |  |  | -1.28  **** | Ref. |
| Af. Am. | AF | -.810  ** | -4.22  **** | .760  ** | -1.50  ** |  |  | .265 | -.995 |  |  | -2.31  **** |  |  |  | -1.27  **** | Ref. |
| Combined | EU+AI | -.242 | -3.46  **** | 1.28  **** | -.446 | .041 | -2.31  **** | .795 | -.540 | -.524 | -.132 | -1.27  **** | -.029 | -.068 | -1.31  **** | -.575  *** | Ref. |
| Combined | EU+AF | -.243 | -3.46  **** | 1.29 **** | -.452 | -.057 | -2.40  **** | .817 | -.509 | -.243 | .152 | -1.02  **** | -.048 | -.000 | -1.02  **** | -.577  *** | Ref. |
| Combined | AI+AF | -.235 | -3.46  **** | 1.31  **** | -.444 | -.076 | -2.42  **** | .842 | -.510 | -.209 | .185 | -.984  **** | -.050 | -.013 | -.985  **** | -.572  *** | Ref |

**Supplementary Table 4 - Beta Coefficients for Sex (Women) and Enrolment Age in the Logistic Regressions for the FIND Study.** Dependent Variable is Diabetic Nephropathy.

| Logistic Model | | Sex | | Enrolment Age | |
| --- | --- | --- | --- | --- | --- |
| **Find**  **Sample** | **IGH**  **Covariate** | Beta | p Value | Beta | p Value |
| Eu. Am. | EU | -0.214 | 0.192 | 0.027 | 0.001 |
| Eu. Am. | AI | -0.204 | 0.213 | 0.027 | 0.002 |
| Eu. Am. | AF | -0.215 | 0.190 | 0.027 | 0.001 |
| Mex. Am. | EU | -0.767 | <0.0001 | 0.012 | 0.039 |
| Mex. Am. | AI | -0.768 | <0.0001 | 0.012 | 0.037 |
| Mex. Am. | AF | -0.755 | <0.0001 | 0.012 | 0.038 |
| Am. Ind. | EU | -0.790 | <0.0001 | 0.015 | 0.042 |
| Am. Ind. | AI | -0.786 | <0.0001 | 0.015 | 0.041 |
| Am. Ind. | AF | -0.783 | <0.0001 | 0.015 | 0.035 |
| Af. Am. | EU | -0.698 | <0.0001 | -0.004 | 0.555 |
| Af. Am. | AI | -0.692 | <0.0001 | -0.004 | 0.545 |
| Af. Am. | AF | -0.698 | <0.0001 | -0.004 | 0.555 |
| Combined | EU+AI | -0.631 | <0.0001 | 0.010 | 0.003 |
| Combined | EU+AF | -0.630 | <0.0001 | 0.010 | 0.003 |
| Combined | AI+AF | -0.629 | <0.0001 | 0.010 | 0.003 |

**Supplementary Table 5 - Distribution of the Differences in Individual Genetic Heritage for Estimates from Two, Independent, Balanced Sets of SNPs: 1300 AIMs in the first and 975 AIMs in the second.**

|  | AIM Sample | | | |
| --- | --- | --- | --- | --- |
| Statistic | HapMap CEU  N=165 | HapMap YRI  N=193 | HapMap LWK  N=110 | Pima  N=964 |
| Mean | -0.003 | -0.002 | 0.002 | 2.0X10^-4^ |
| Standard Deviation | 0.016 | 0.010 | 0.020 | 0.008 |
| Median | 0.000 | 0.000 | 0.002 | 0.000 |
| Mode | 0.000 | 0.000 | 0.000 | 0.000 |
