# Supplementary material for: Selecting SNPs informative for African, American Indian and European Ancestry: application to the Family Investigation of Nephropathy and Diabetes (FIND)
Source: BMC Genomics. 2016 May 4;17:325. doi: 10.1186/s12864-016-2654-x (PMC4855449; doi:10.1186/s12864-016-2654-x)
Supplement: Additional file 5: Figure S1. — Repeating the individual admixture estimates using the STRUCTURE program (K=3). Figure S2. STRUCTURE estimates for four ancestral populations with 400 SNPs maximized for the American Indian-African contrast. Figure S3. STRUCTURE estimates for four ancestral populations with 450 SNPs maximized for the European-American Indian contrast. Figure S4. STRUCTURE estimates for four ancestral populations with 450 SNPs maximized for the European-African contrast. Figure S5. STRUCTURE estimates for four ancestral populations with all 1300 SNPs with balanced information for each contrast. (DOCX 1104 kb) [file 12864_2016_2654_MOESM5_ESM.docx]

Supplementary Figure 1


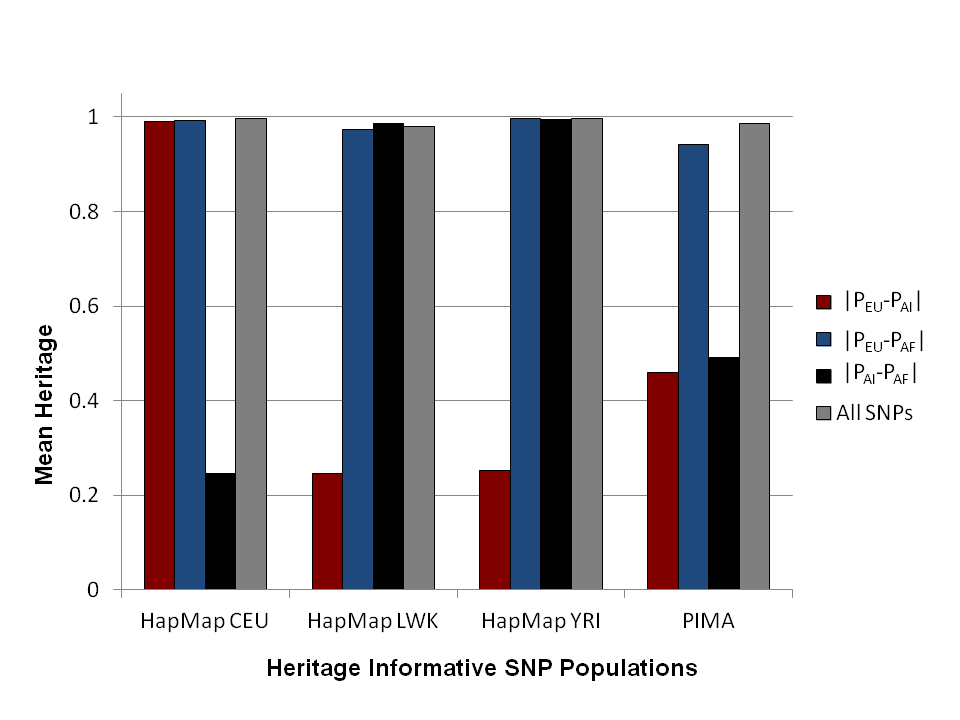


**Supplementary Figure 1 - Repeating the individual admixture estimates using the STRUCTURE program (K=3).** It gave similar results to the fixed parental allele frequency algorithm but showed, in addition, that it was even more sensitive to imbalances in information. It did not return the expected mean value of AI for Pima Indians even when the contrast, |P_AI_-P_AF_| or |P_EU_-P_AI_|, was maximized for this component (Supplementary Table 1); the fixed parental allele algorithm always returned the correct mean expected values for the components maximized in the contrast when all 3 components were being simultaneously estimated (Table 4, Figure 2). When the Bayesian cluster algorithm was used with all 1300 SNPs with balanced information, it returned the appropriate mean expected values for all ancestry samples.

Supplementary Figure 2


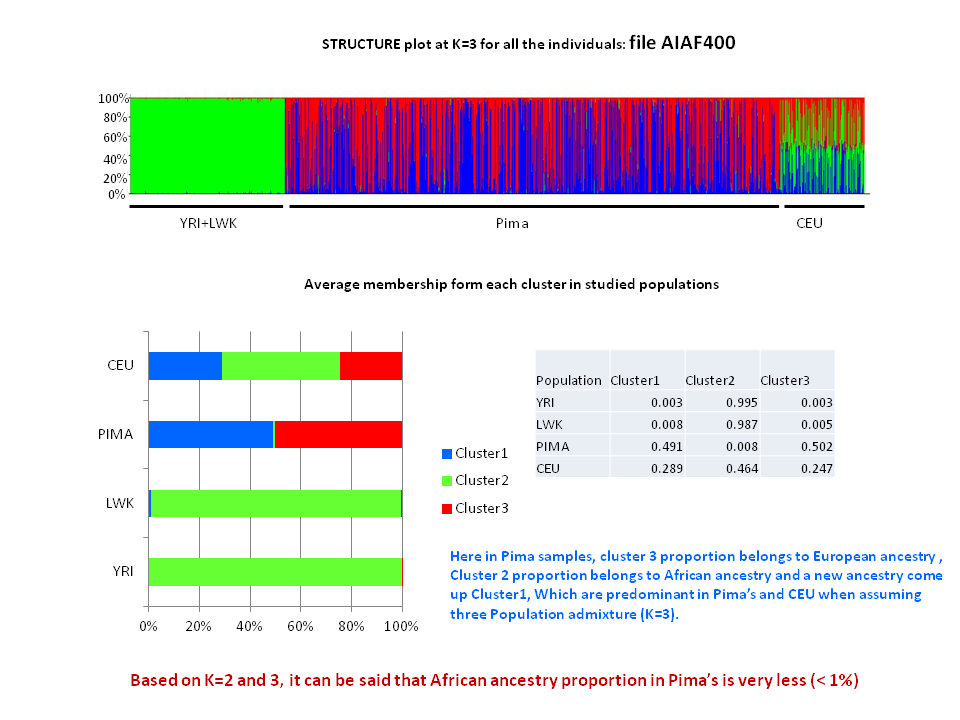


**Supplementary Figure 2: STRUCTURE Estimates for Four Ancestral Populations with 400 SNPs Maximized for the American Indian-African Contrast.** The STRUCTURE program returns the correct expected mean of ancestral component for the African ancestral populations LWK and YRI (Cluster 2) but does not return a mean of approximately 1.0 for the American Indian component even though this contrast is maximized for it. Given little information for the European component in this set of SNPs, the estimates appear randomly assigned.

Supplementary Figure 3


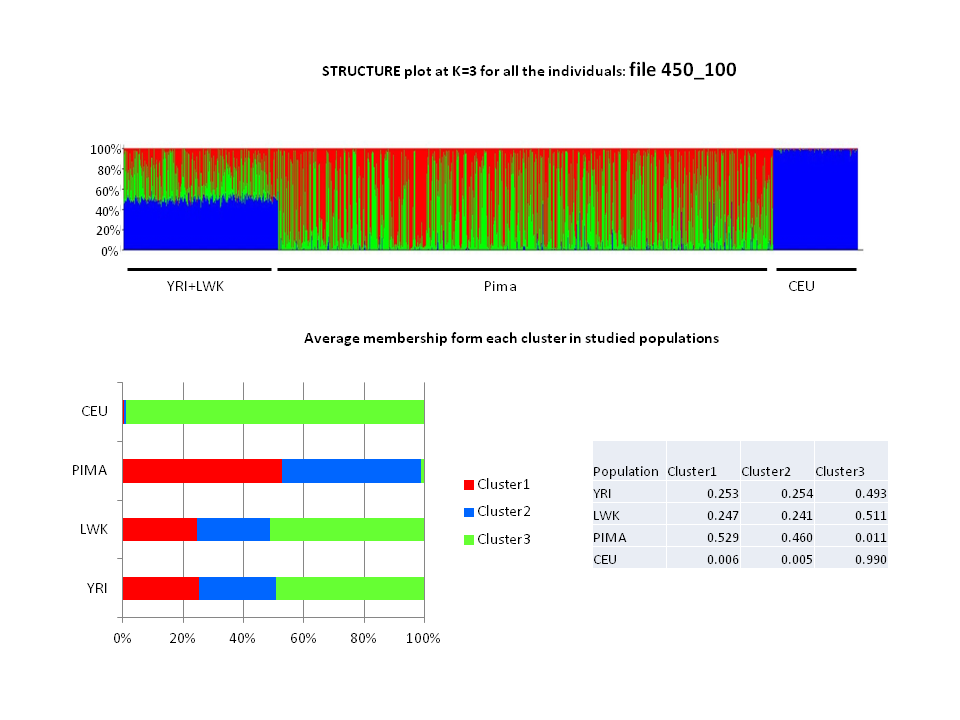


**Supplementary Figure 3: STRUCTURE Estimates for Four Ancestral Populations with 450 SNPs Maximized for the European-American Indian Contrast.** The STRUCTURE program returns the correct expected mean of ancestral component for the European ancestral population CEU (Cluster 3) but does not return a mean of approximately 1.0 for the American Indian component even though this contrast is maximized for it. Given little information for the African component in this set of SNPs, the estimates appear randomly assigned for LWK and YRI.

Supplementary Figure 4


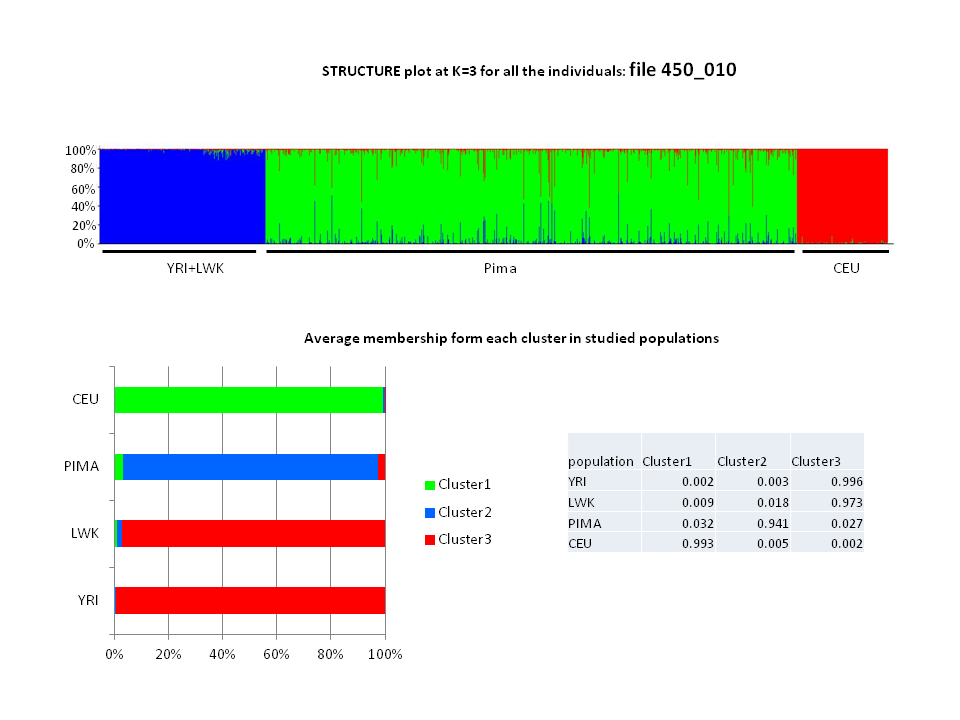


**Supplementary Figure 4: STRUCTURE Estimates for Four Ancestral Populations with 450 SNPs Maximized for the European-African Contrast.** The STRUCTURE program returns the correct expected mean of ancestral component for the African ancestral populations LWK and YRI (Cluster 3) and also for the European component (Cluster 1). The surprising result for this run is that the Pima component for Cluster 2 also approximates the expected mean of 1.0 even though this contrast is not maximized for American Indian information.

Supplementary Figure 5


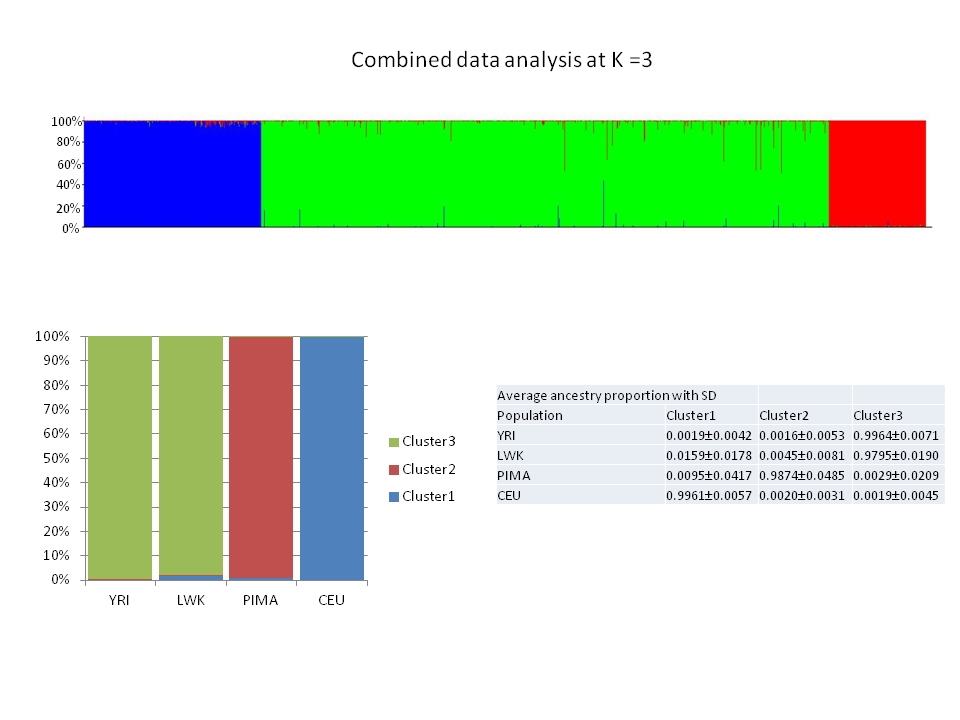


**Supplementary Figure 5: STRUCTURE Estimates for Four Ancestral Populations with all 1300 SNPs with Balanced Information for each Contrast.** With the set of 1300 SNPs and information balanced across the 3 contrasts the program returns the correct expected mean of ancestral ancestry for all components. Cluster 1 is European ancestry; Cluster 2 is American Indian ancestry; and Cluster 3 is African ancestry.
